# Optogenetic activation of mesencephalic projections to the nucleus accumbens shell impairs probabilistic reversal learning by disrupting learning from negative reinforcement

**DOI:** 10.1111/ejn.16584

**Published:** 2024-10-31

**Authors:** Katharina Zühlsdorff, Júlia Sala‐Bayo, Sammy Piller, Peter Zhukovsky, Thorsten Lamla, Wiebke Nissen, Moritz von Heimendahl, Serena Deiana, Janet R. Nicholson, Trevor W. Robbins, Johan Alsiö, Jeffrey W. Dalley

**Affiliations:** ^1^ Department of Psychology University of Cambridge Cambridge UK; ^2^ Boehringer Ingelheim Pharma GmbH & Co. KG, Div. Research Germany Biberach an der Riß Germany; ^3^ Department of Psychiatry, Herchel Smith Building for Brain & Mind Sciences University of Cambridge Cambridge UK; ^4^ Present address: School of Physiology, Pharmacology and Neuroscience University of Bristol Bristol UK

**Keywords:** cognitive flexibility, dopamine, negative feedback, optogenetics, striatum

## Abstract

Cognitive flexibility, the capacity to adapt behaviour to changes in the environment, is impaired in a range of brain disorders, including schizophrenia and Parkinson's disease. Putative neural substrates of cognitive flexibility include mesencephalic pathways to the ventral striatum (VS) and dorsomedial striatum (DMS), hypothesized to encode learning signals needed to maximize rewarded outcomes during decision‐making. However, it is unclear whether mesencephalic projections to the ventral and dorsal striatum are distinct in their contribution to flexible reward‐related learning. Here, rats acquired a two‐choice spatial probabilistic reversal learning (PRL) task, reinforced on an 80%|20% basis (correct|incorrect responses) that assessed the flexibility of behaviour to repeated reversals of response‐outcome contingencies. We report that optogenetic stimulation of projections from the ventral tegmental area (VTA) to the nucleus accumbens shell (NAcS) in the VS significantly impaired reversal learning when optical stimulation was temporally aligned with negative feedback (i.e., reward omission). VTA → NAcS stimulation during other phases of the behavioural task was without significant effect. Optogenetic stimulation of projection neurons from the substantia nigra (SN) to the DMS, aligned either with reward receipt or omission or prior to making a choice, had no significant effect on reversal learning. These findings are consistent with the notion that increased activity in the VTA → NAcS pathway disrupts behavioural flexibility by impairing learning from negative reinforcement.

## Graphical abstract

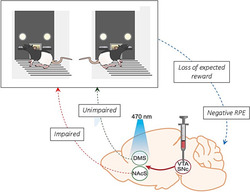

**Abbreviations**
AL: after loss
AP: anteroposterior
ASL: after spurious loss
AW: after loss
AWS: after spurious win
DA: dopamine
DMS: dorsomedial striatum
DV: dorsoventral
ITI: intertrial interval
LH: limited hold
ML: mediolateral
NAcC: nucleus accumbens core
NAcbS: nucleus accumbens shell
NGS: normal goat serum
PBS: phosphate‐buffers saline
PD: Parkinson's disease
PFA: paraformaldehyde
PRL: probabilistic reversal learning task
RPE: reward prediction errors
SN: substantia nigra
TH: tyrosine hydroxylase
TO: time‐out
TTC: trials to criterion
UUC: up until choice
VS: ventral striatum
VTA: ventral tegmental area

## 1 INTRODUCTION

Cognitive flexibility refers to the capacity of individuals to shift behaviour adaptively to optimize rewarded outcomes. Flexible responding requires dynamic updating of value associated with stimuli and/or actions (O'Doherty, 2011) and depends in part on signalling from midbrain dopamine (DA) neurons (Cools et al., 2001, 2007). The ability to switch behaviour adaptively is compromised in a broad range of neurological and neuropsychiatric disorders, including Parkinson's disease (PD) (Cools et al., 2001), schizophrenia (Leeson et al., 2009) and obsessive‐compulsive disorder (Remijnse et al., 2013).

Supporting a role for DA in cognitive flexibility, depletion of DA in the caudate nucleus of monkeys (Clarke et al., 2011) or homologous dorsomedial striatum (DMS) of rats (O'Neill & Brown, 2007) impaired the flexible reversal of previously learnt stimulus–reward associations. In contrast, local DA receptor activation in the ventral striatum impaired reversal learning (Verharen et al., 2019), while infusing DA receptor antagonists into the nucleus accumbens shell (NAcS) or core (NacC) facilitated reversal learning (Sala‐Bayo et al., 2020). Such findings support the hypothesis that DA neurotransmission in the DMS and nucleus accumbens (NAc) exerts opposing effects on reversal learning. Such divergence may underlie the impairments in probabilistic reversal learning (PRL) produced by L‐DOPA and other DA medications in PD patients (Cools et al., 2001; Frank et al., 2004). Thus, the same PD medications that restore DA levels in the nigrostriatal system where signalling is reduced also boost DA levels in the mesolimbic system where DA signalling is less affected. This may lead to ‘DA overdose’ in the mesolimbic system and cause adverse effects of DA replacement therapy, including impairments in cognitive flexibility (Cools, 2006).

Midbrain DA projections from the ventral tegmental area (VTA) and substantia nigra pars compacta (SNc) to the striatum support the coding of both positive and negative reward prediction errors (RPEs), specifically the signalling of discrepancies between expected and received rewards and thereby value‐based learning of stimuli associated with actions (Chang et al., 2015; Schultz, 1998; Steinberg et al., 2013). Consistent with this notion, chemogenetic activation of the VTA → NAc pathway impaired spatial reversal learning in rats, an effect linked to learning from reward omissions (Verharen et al., 2018). However, few studies have investigated how RPEs causally affect associative learning in the context of reversal learning, despite a growing number of studies reporting temporally aligned neuronal signalling and stimulus–reward learning (Aquili, 2014; Chang et al., 2015; Steinberg et al., 2013).

**FIGURE 1:**
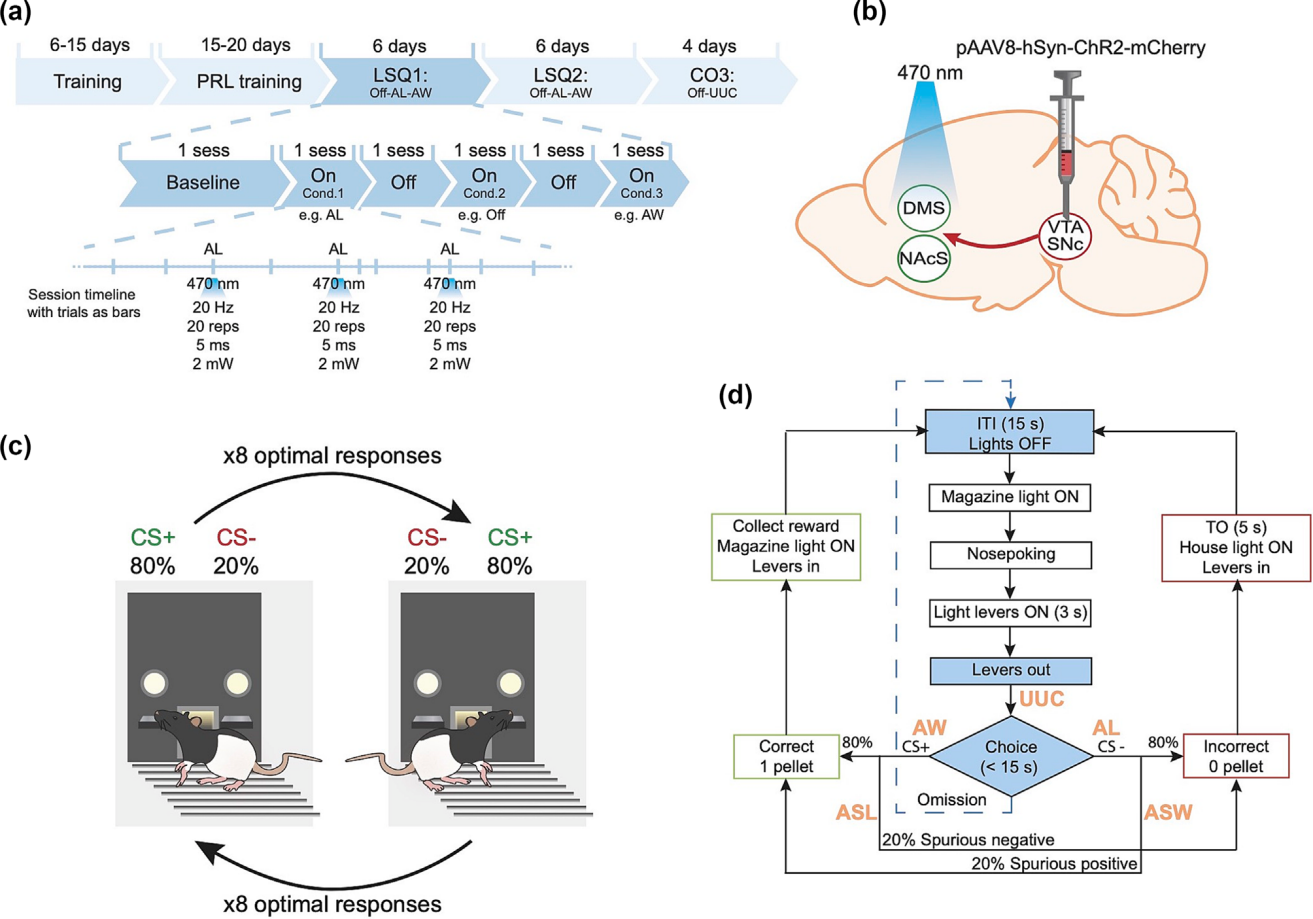
Summary of the experimental procedures used for in vivo optogenetic stimulation of the mesoaccumbal and nigrostriatal pathways during a spatial probabilistic reversal learning (PRL) task. (a) Timeline of the behavioural procedure and optical stimulation. Following pretraining stages to respond to the levers, animals were trained in the PRL task. In later sessions of this stage, animals were connected to the patch‐cord cables without light stimulation. Once performance had stablilized (i.e., ≥3 reversals in three sessions in a row), testing started. Testing consisted of two Latin‐square and one crossover (LSQ; CO) experiment. A LSQ is an experiment that controls for the order of presentation through counterbalancing. A CO experiment is a design where subjects are switched between different treatment groups to serve as their own controls. Each LSQ started with a baseline session consisting of a PRL session with the cables connected to the rats, without light stimulation. The different conditions (e.g., off‐AL‐AW) were then tested. Light (λ = 470 nm) was selectively turned on after each trial that presented the tested condition, for example, after not receiving a reward (AL). Intracranial stimulation was induced with 20 repetitions of 5‐ms pulses, with 2 mW at the tip of the optical fibres. Testing conditions within each LSQ or CO experiment were pseudo‐randomized. Final group sizes: DMS (LSQ1) *n* = 18, (LSQ2) *n* = 15, (CO3) *n* = 26; and NAcS (LSQ1) *n* = 15, (LSQ2) *n* = 13, (CO3) *n* = 13. Abbreviations: after loss (AL), after win (AW), after a spurious loss (ASL), after a spurious win (ASW), up until making a choice (UUC), session (sess). (b) Schematics showing viral vector infusion in the ventral tegmental area (VTA)/nigra pars compacta (SNc) and fibreoptic implantation in the NAcS or DMS. (c) Illustrative overview of the PRL task in lever‐pressing chambers. After eight consecutive correct trials on the optimal lever (CS+; 80% rewarded), the contingencies were reversed such that the previously optimal lever was now suboptimal (CS‐; 20% rewarded), and vice versa. (d) Flowchart of the PRL task. Shown in orange are the time points where neuronal pathways were optogenetically stimulated for the following events: AL, AW, ASL, ASW and UUC.

Here, we sought to demonstrate a causal link between midbrain neuronal activity and reversal learning performance in rats by dissociating the effects of positive and negative feedback during PRL. Reward on this task was delivered on 80% of correct trials and 20% of incorrect trials (see Figure 1). Thus, to maximize food reward, animals were required to discount spurious negative and positive feedback. We used in vivo optogenetics to investigate the effects of activating either the VTA → NAcS pathway or the medial SNc → to DMS pathway on reversal learning performance. We focus on the DMS rather than the dorsolateral striatum, as the former has been implicated as a key region involved in serial reversal under probabilistic uncertainty (Lhost et al., 2021; Young et al., 2023). Specifically, we determined the impact on reversal learning of activation of the VTA → NAcS and medial SNc → to DMS pathways after each of the following events: (1) the delivery of reward following a correct response; (2) the omission of reward following a correct response; (3) the delivery of reward following an incorrect response; (4) the omission of reward following an incorrect response. We also investigated the effects on reversal learning of activation of the two pathways originating from the mesencephalon immediately before the selection of a response (i.e., after the presentation of the response levers).

We hypothesized that increased VTA‐NAcS neuronal activation timed to coincide with reward omission (i.e., negative RPE) would impair reversal learning by interfering with the putative endogenous dip in DA release that encodes the teaching signal to omitted rewards (Schultz et al., 1997). We predicted that this intervention would decrease shifting behaviour and the rate of learning from unrewarded or negative feedback trials. We further predicted that increasing VTA‐NAcS neuronal activation on rewarded trials, thereby mimicking positive RPEs, would either enhance reversal performance by amplifying the presumed natural burst of DA release or would remain unaltered due to possible ceiling effects. Given the opponent effects of dorsal versus ventral DA signalling on reversal learning, discussed above, we anticipated that SNc → DMS neuronal activation would result in broadly opposite effects on reversal learning compared with VTA → NAcS pathway activation. We report that activation of the VTA‐NAcS pathway but not the SNc → DMS pathway impairs reversal learning when stimulation occurs at the time of reward omission.

## 2 MATERIALS AND METHODS

### 2.1 Subjects

Forty‐six male Lister‐Hooded rats (Charles River, Germany) were initially housed in groups of four under humidity‐ and temperature‐controlled conditions and a 12/12‐h light–dark cycle (lights off at 07:30 h). Rats were maintained at 90% of their free‐feeding weight by food restriction. Water was provided ad libitum. *E* *xperiments were aligned with the German animal welfare legislation, Association for Assessment and Accreditation of Laboratory Animal Care regulations and approved by the Local Animal Care and Use Committee*.

### 2.2 Stereotaxic surgery

Anaesthesia was induced with 5% isoflurane in oxygen and maintained at 2.5%. Rats were secured in a stereotaxic frame fitted with atraumatic ear bars (KOPF Model 1900, Germany). The skull surface was manually cleaned after a midline incision dorsally, and OptiBond® All‐in‐One bone glue (Kerr, USA) was applied and hardened with UV light for 60 s. Animals were bilaterally infused with a maximal total volume of 2400 nL of viral vector, divided across four infusions (two infusions per hemisphere) at a flow rate of 200 nL/min. Recombinant AAVs (rAAV) were produced by transient transfection of adherently grown HEK‐293H cells in CELLdiscs and purified by PEG‐precipitation, iodixanol density gradient ultracentrifugation, Amicon‐15 ultrafiltration and sterile filtration (Strobel et al., 2015, 2019). Genomic titres were determined by qPCR using primers specific to the human synapsin promoter sequence. Optogenetic animals received opsin‐expressing rAAV8‐hSyn‐ChR2‐mCherry, whereas control animals received rAAV8‐hSyn‐mCherry. Animals were divided into two groups: (1) VTA → NAcS; (2) SNc → DMS. For the first group, the virus was infused into the VTA at anteroposterior (AP) −5.4 and −6.2; mediolateral (ML) ±0.6, dorsoventral (DV) −8.4 and −7.8 and optical fibres (Doric Lenses, Canada) were implanted in the NAcS at AP +1.5, ML ±0.8, DV –7.0. For the second group, the virus was infused into the SNc at AP −5.4, ML ±0.6; DV −8.1 and −8.0, and optical fibres (Doric Lenses, Canada) were implanted in the DMS at AP +1.2, ML ±2.0, DV –5.3 (Figure 1b). Coordinates refer to millimeters from Bregma and the skull surface. Implants were secured with dental cement, four skull screws and dental product Charisma® (Kulzer, Germany) and hardened with UV light for 20 s. Virus injections and optical fibre implantations were performed bilaterally. All surgeries took place at least 4 weeks before the start of behavioural testing. Behavioural training commenced 7 days after surgery.

### 2.3 Behavioural training

Rats were trained in eight operant chambers (Med Associates, Georgia, VT, USA), the setup of which is shown in Figure 1c. Behavioural training in the PRL task was modified from Bari et al. (2010) for the use of retractable levers instead of nose‐poking holes. Briefly, the first stage consisted of a 60 min or 40 trial session, whichever came first. When one of the two levers was pressed, three 45 mg sucrose pellets (5TUL, TestDiets, USA) were delivered, and both levers were withdrawn; if no lever was pressed within 30 s, one single pellet was delivered, and both levers were withdrawn. An omitted trial did not count towards the completed trials. The criterion to move to the next stage was the completion of all 40 trials.

The following training stages consisted of sessions of 60 min or 120 trials, whichever came first and incorporated an intertrial interval (ITI) of 10 s, a time‐out (TO) of 5 s after an incorrect response and a limited hold (LH) of 30 s, after which levers were retracted and the trial was deemed an omission. The criterion to move to subsequent stages was the attainment of ≥80 correct trials. In Stage 2, animals were trained to press either lever to receive a reward. After 60 trials, the preferred lever was retracted to force animals to press the opposite lever. In Stage 3, animals had to nose poke an empty magazine to initiate each trial. In Stage 4, rats learned that levers were rewarded in a probabilistic manner. For this, both cue lights were illuminated for 3 s, after which only one of the levers was extended, which was rewarded on 80% of the trials. After 30 consecutive trials, the opposite lever was extended for the next 30 trials, and so on until the rat had completed 120 trials. At least two sessions reaching criterion were required to move to PRL task training.

### 2.4 PRL task

Daily sessions consisted of 200 trials or 60 min, whichever came first. At the start of each session, one of the two levers was randomly selected to be the ‘optimal’ lever. Sessions began with illumination of the magazine light. After nose poking, the magazine light was extinguished, and the two cue lights turned on, indicating that the levers would be available 3 s later. A response to the optimal lever delivered a single reward pellet on 80% of trials, whereas a response to the suboptimal lever yielded reward on only 20% of trials. A failure to press any lever was noted as an omission. After eight consecutive correct trials (i.e., pressing the ‘optimal’ lever regardless of it being reinforced or not), the contingencies were reversed (Figure 1c). Animals were trained until they could achieve at least three reversals per session over three consecutive sessions. Once this criterion was met, rats underwent testing.

### 2.5 Behavioural testing

Rats received a minimum of two habituation training sessions with the cables attached to their implant. A baseline session always occurred on the day before testing with the cables attached but with no optical stimulation. Rats were randomly assigned to an optogenetic stimulation group or a ‘light‐off’ group (Figure 1d). The optogenetic stimulation conditions were (1) ‘after loss’ (AL) when pressing the suboptimal lever; (2) ‘after win’ (AW), when pressing the optimal lever; (3) ‘after spurious loss’ (ASL) when pressing the optimal lever but not receiving the expected reward (20% of the times); (4) ‘after a spurious win’ (ASW) when pressing the suboptimal lever but receiving an unexpected reward (20%); and (5) ‘up until choice’ (UUC) from the start of the trial (presentation of the levers) until pressing a lever (Figure 1d). All conditions were pseudo‐randomized according to baseline levels of performance using a Latin‐square design (LSQ; (1) Off—AL—AW; (2) Off—ASL—ASW), or cross‐over design (CO; (3) Off—UUC) (Figure 1a). Testing took place every second day. On intervening days, animals were run with cables attached but with the light off to maintain stable levels of performance and to avoid possible carryover effects of light stimulation.

### 2.6 Optical stimulation

Mono‐fibreoptic patch‐cord cables (Doric Lenses, Canada) were metal shielded and terminated in an optical fibre of 200 μm diameter, and a numerical aperture of 0.37. One end of the cable was connected to a dual LED commutator via magnetic compact LED modules (Plexon, Dallas TX, USA). The other end of the cable was secured to the rats' implants with a fitted zirconia sleeve (Doric Lenses, Canada). A computer running Med Associates software, which also recorded responses, controlled the optical stimulation. A second computer controlled the behavioural task via transistor‐transistor logic signals.

Patch‐cord cables were covered with 16 cm plastic tubes. Cables were secured to the rats' implants with a zirconia sleeve (Doric Lenses, Canada) for a 1.25 mm diameter ferrule. Intracranial stimulation was achieved with 20 repetitions of 5‐ms light pulses (20 Hz), delivering 2 mW at the tip of each optical fibre (Figure 1a). Data from sessions where light output was compromised because of broken or disconnected optical cables were discarded. Final group sizes were DMS (LSQ1) *n* = 26, (LSQ2) *n* = 24, (CO3) *n* = 20; NAcS (LSQ1) *n* = 14, (LSQ2) *n* = 15, (CO3) *n* = 11.

### 2.7 Histological assessment of fibre‐optic probe placement and viral vector expression

Following completion of the behavioural procedures, animals were anaesthetized with a lethal dose of pentobarbital (Boehringer Ingelheim GmbH, Germany) and perfused transcardially with 0.01 M phosphate‐buffers saline (PBS) followed by 4% paraformaldehyde (PFA). Brains were removed and post‐fixed in 4% PFA for 24 h and dehydrated for cryoprotection in 30% sucrose in 0.01 M PBS.

Brains were coronally sectioned at 60 μm using a cryostat (Leica, Germany), collected in PBS containing 25% polyethylene glycol and 25% glycerol and stored at 4°C. Free‐floating sections were washed in PBS and subsequently blocked and permeabilized in PBS containing 3% normal goat serum (NGS) and 0.3% Triton for 1 h. Sections were incubated overnight with primary antibodies in PBS containing 3% NGS and 0.3% Triton. Since the infused viral vector inherently expressed fluorescence, no antibodies were required to detect transgene expression. For the first set of slices, tyrosine hydroxylase (TH) was detected with the primary antibody anti‐TH in rabbit (1:600, EMD Millipore, USA). After washing in PBS, sections were incubated with secondary antibodies for 2 h (anti‐rabbit in goat Alexa‐Fluor 488 nm, 1:500, Invitrogen Thermo Fisher Scientific, USA). For the second set of slices, double staining was achieved with the primary antibodies anti‐GAD67 in mouse (1:600, Invitrogen Thermo Fisher Scientific, USA) and anti‐VGLUT2 in rabbit (1:600, Invitrogen Thermo Fisher Scientific, USA). Secondary antibodies were goat anti‐mouse (Alexa‐Fluor 647 nm, 1:500, Invitrogen Thermo Fisher Scientific, USA) and goat anti‐rabbit (Alexa‐Fluor 488 nm, 1:500, Invitrogen Thermo Fisher Scientific, USA). After washing in PBS, sections were mounted in distilled water and covered with mounting medium (DAPI, EMD Millipore, USA) and a coverslip. Immunofluorescence sections were checked and digitized using a PerkinElmer Opera Phenix High‐Contrast Screening microscope (PerkinElmer, USA). Fibre counting was performed in the NAcS and DMS.

### 2.8 Behavioural data analysis

The following behavioural measures were extracted and analysed: number of trials to reach a contingency change (TTC), where the criterion is eight consecutive correct responses, number of reversals, proportion correct responses, percentage of lose‐shift behaviour after a correct or incorrect response, percentage of win‐stay after a correct or incorrect response and the average number of perseverative responses after a reversal.

Statistical tests were performed using R version 4.0.4 (R Core Team, 2021). Data were subjected to linear mixed‐effects model analysis with the lmer package (Bates et al., 2015). The model contained two fixed factors (experiment—e.g., after win/after loss, light—on/off) and one factor (subject) modeled as an intercept to account for individual differences between rats. Outliers were identified using Tukey's method, which highlights outliers ranged above and below 1.5* the interquartile range using the *outlierKD* function (Klodian, 2017). The identified outliers were removed from subsequent analyses. When significant interactions were found, further analyses were carried out using post hoc pairwise comparisons using the *emmeans* package (Lenth et al., 2018). Significance was considered at *p* < 0.05.

## 3 RESULTS

### 3.1 Histological analysis

**FIGURE 2:**
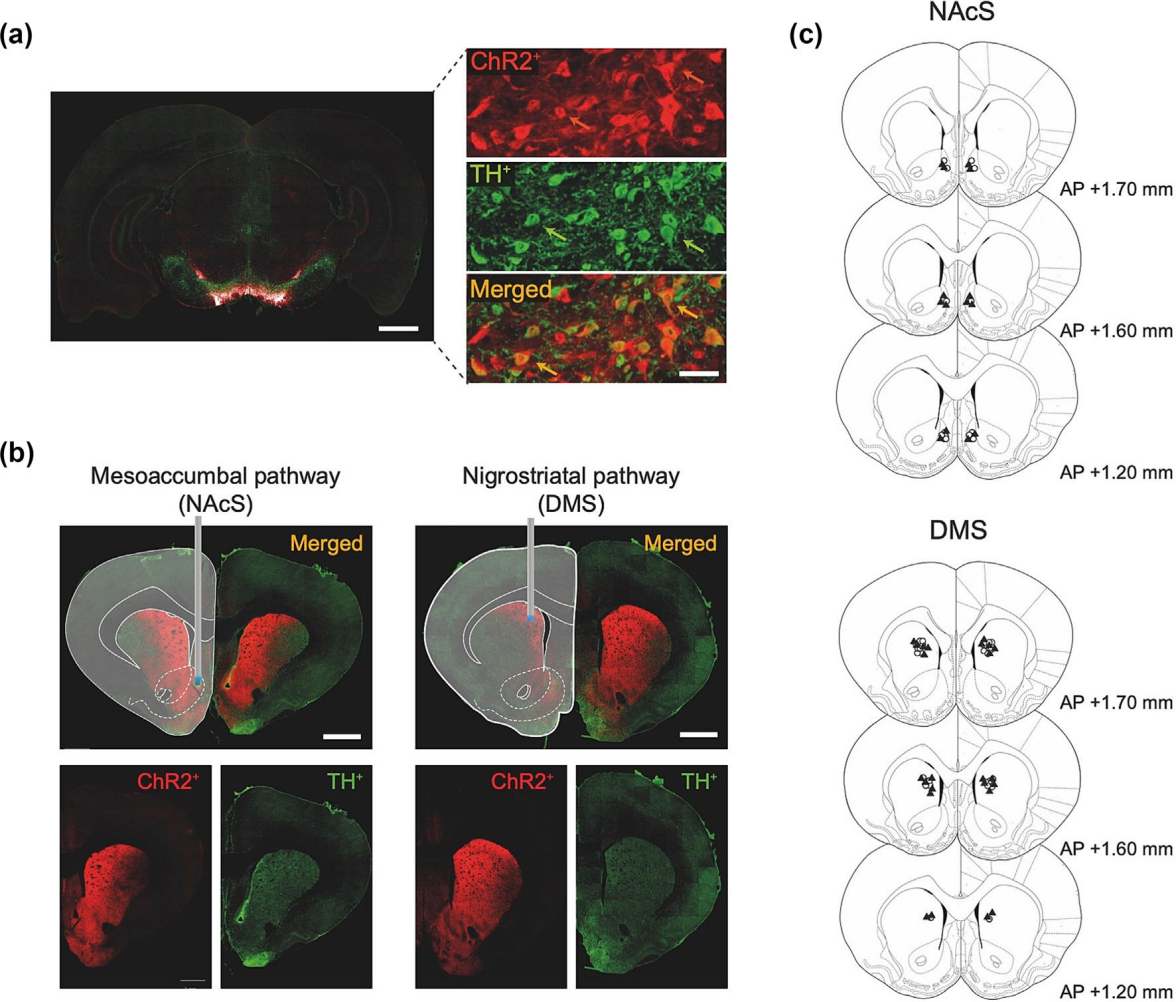
Histological analysis showing viral expression and fibreoptic placements in the nucleus accumbens shell and dorsomedial striatum. (a) (left) coronal section of the ventral tegmental area (VTA) stained for tyrosine hydroxylase (TH) showing viral‐transfected neurons. Scale bar: 1 mm. (right top) detailed expression of virus positive neurons, (right middle) TH^+^ neurons and (right bottom) both channels merged. Scale bar: 50 μm. (b) Representative histology images showing coronal sections of the striatum and representative fibre optical tip location in the NAcS (left) and DMS (right). Expression of viral vector (ChR2; bottom left), TH (bottom right) and both channels merged (top). Scale bars: 1 mm. (c) Fibre optic tip placements in the NAcS and DMS. Full triangles: opsin group. Empty circles: control group. Anteroposterior (AP) coordinates from Bregma.

Figure 2a,b shows that administration of opsin‐expressing virus into the VTA or SNc resulted in expression of ChR2 in both the NAcS and the DMS, respectively. Figure 2c shows fibreoptical tip placements in the NAcS or DMS for those animals that completed the study.

The expression of virus and neuronal markers in the neuronal fibres was measured in the DMS and NAcS to quantify the neurochemical phenotype of infected neurons; 55.28% ± 3.74% of transfected neurons in the NAcS were TH positive (*n* = 5), 15.24% ± 1.56% were VGLUT positive (*n* = 7), and 4.77% ± 1.13% were GAD67 positive (*n* = 7). In the DMS, 73.51% ± 2.26% were TH positive (*n* = 9), 8.99% ± 0.69% were VGLUT2 positive (*n* = 7), and 6.17% ± 0.83% were GAD67 positive (*n* = 7).

### 3.2 Behavioural results

#### 3.2.1 Activation of the VTA‐NAcS pathway impairs reversal learning after reward omission

**FIGURE 3:**
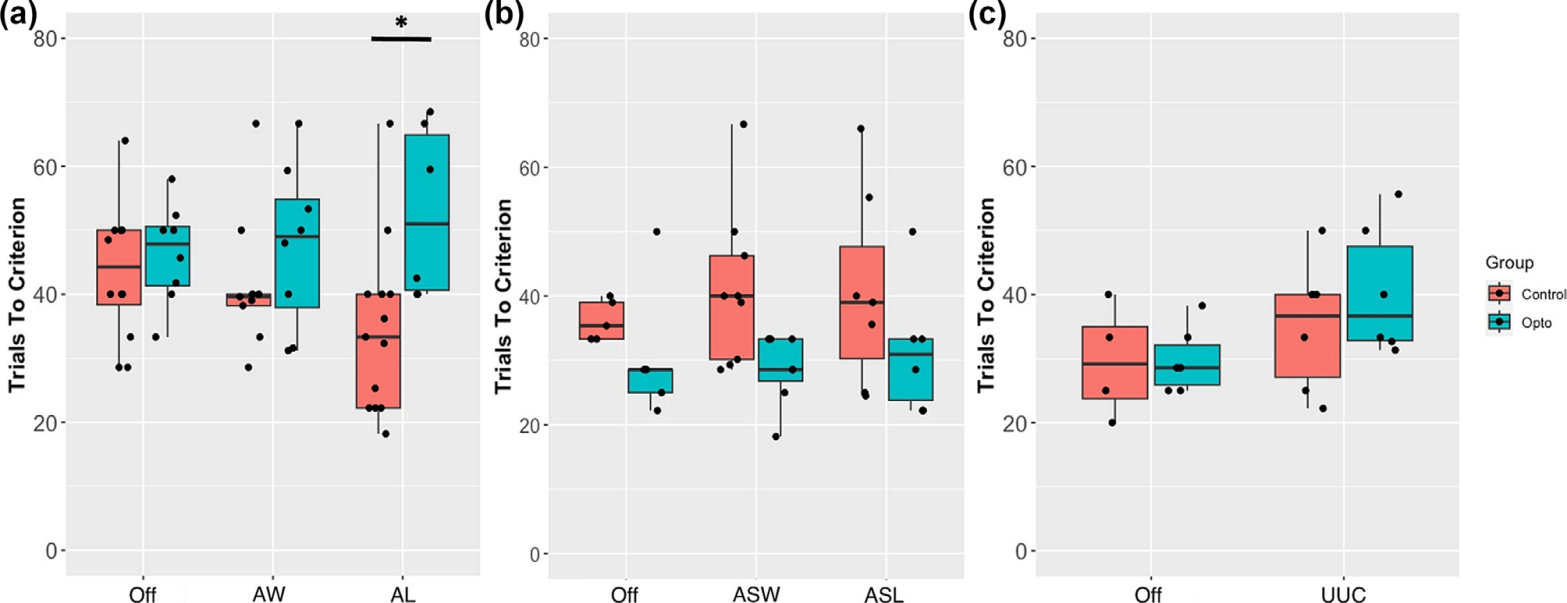
In vivo optogenetic stimulation of the mesoaccumbal pathway impairs reversal learning when selectively activated after the loss of reward. Trials required to reach eight subsequent correct responses in each experiment: after win/after loss (*n* = 15) (a), after spurious win/after spurious loss (*n* = 13) (b), up until choice (*n* = 11) (c). Trials to criteria significantly increased in the opsin group (vs. the control group) following reward omission (*p < 0.05). The bars represent the range of data within 1.5 times the IQR from the lower and upper quartiles. AL, after loss; ASL, after spurious loss; AW, after win; ASW, after spurious win; Off, off light; UUC, up until choice.

Optogenetic stimulation of the VTA‐NAcS pathway was delivered at different time points during the PRL task. Since the *p*‐value for the interaction between opsin group and stimulation condition, with respect to the AL/AW experiment, was less than 0.1 (F_2,42_ = 3.07, *p* = 0.057, η_p_^2^ = 0.08), post hoc analyses were carried out according to the guidance of Midway et al. (2020). This analysis revealed that optogenetic stimulation of the VTA‐NAcS pathway selectively impaired performance by increasing the number of trials required for a contingency change to occur compared with the no‐light condition when optogenetic stimulation was presented after the loss of reward (t_36_ = −3.05, *p* = 0.045; Figure 3) but not after reward delivery. Moreover, there was a significant group × condition interaction with respect to win‐stay behaviour after a correct response (F_2,45_ = 3.35, *p* = 0.044, η_p_^2^ = 0.13). However, following correction for multiple comparisons, no post hoc tests were significant (Table 1). Optical stimulation had no significant effect on the proportion of correct responses, lose‐shift behaviour or perseverative responses when applied after spurious reward losses or spurious reward wins compared with the light‐off condition (i.e., Off vs. ASL or Off vs. ASW) or when applied before a response was selected (i.e., Off vs. UUC).

**TABLE 1:** Summary of win‐stay and lose‐shift probabilities across all experiments and groups.

		LSQ1						LSQ2						CO3			
Group		Control			Opto			Control			Opto			Control		Opto	
Condition		*Off light*	*AW*	*AL*	*Off light*	*AW*	*AL*	*Off light*	*ASW*	*ASL*	*Off light*	*ASW*	*ASL*	*Off light*	*UUC*	*Off light*	*UUC*
Experiment—Win‐stay	**SN—DMS**	0.77 ± 0.019	0.77 ± 0.016	0.75 ± 0.032	0.72 ± 0.015	0.78 ± 0.011	0.71 ± 0.023	0.81 ± 0.016	0.81 ± 0.022	0.79 ± 0.020	0.80 ± 0.011	0.79 ± 0.023	0.77 ± 0.017	0.83 ± 0.019	0.82 ± 0.014	0.82 ± 0.023	0.79 ± 0.017
	**VTA—NAcS**	0.73 ± 0.012	0.79 ± 0.025	0.80 ± 0.017	0.77 ± 0.029	0.73 ± 0.026	0.75 ± 0.019	0.80 ± 0.014	0.76 ± 0.016	0.77 ± 0.029	0.80 ± 0.032	0.79 ± 0.035	0.78 ± 0.028	0.83 ± 0.034	0.80 ± 0.031	0.80 ± 0.022	0.77 ± 0.026
Experiment—Lose‐shift	**SN‐DMS**	0.44 ± 0.015	0.44 ± 0.015	0.46 ± 0.016	0.47 ± 0.013	0.45 ± 0.019	0.46 ± 0.013	0.47 ±0 .015	0.044 ± 0.017	0.47 ± 0.015	0.48 ± 0.012	0.46 ± 0.021	0.50 ± 0.017	0.46 ± 0.016	0.45 ± 0.017	0.48 ± 0.017	0.49 ± 0.016
	**VTA—NAcS**	0.46 ± 0.020	0.46 ± 0.034	0.46 ± 0.026	0.52 ± 0.021	0.54 ± 0.023	0.49 ± 0.019	0.46 ± 0.040	0.53 ± 0.027	0.46 ± 0.035	0.43 ± 0.028	0.42 ± 0.020	0.44 ± 0.018	0.46 ± 0.015	0.44 ± 0.021	0.52 ± 0.021	0.47 ± 0.025

*Note*: Values given are means ± one standard error of the mean.

Abbreviations: DMS, dorsomedial striatum; SN, substantia nigra; VTA, ventral tegmental area.

With respect to the AL/AW experiment, a significant group × condition interaction was observed with respect to response latencies (F_2,42_ = 3.52, *p* = 0.039, η_p_^2^ = 0.11). Response latencies were slower when optostimulation was applied after a win compared to after a loss in the opsin group (t_42_ = −2.71, *p* = 0.044). However, for the remaining two experiments (ASL/ASW and UUC), no significant effects were present for either response or collection latencies (Table 2).

**TABLE 2:** Summary of response and collection latencies across all experiments and groups.

		LSQ1						LSQ2						CO3			
Group		Control			Opto			Control			Opto			Control		Opto	
Condition		*Off light*	*AW*	*AL*	*Off light*	*AW*	*AL*	*Off light*	*ASW*	*ASL*	*Off light*	*ASW*	*ASL*	*Off light*	*UUC*	*Off light*	*UUC*
Experiment – Response latencies	**SN—DMS**	0.35 ± 0.019	0.34 ± 0.025	0.34 ± 0.029	0.36 ± 0.019	0.31 ± 0.016	0.33 ± 0.023	0.33 ± 0.016	0.33 ± 0.024	0.33 ± 0.025	0.35 ± 0.19	0.32 ± 0.021	0.32 ± 0.020	0.31 ± 0.018	0.32 ± 0.024	0.33 ± 0.021	0.34 ± 0.019
	**VTA—NAcS**	0.29 ± 0.019	0.28 ± 0.023	0.30 ± 0.023	0.37 ± 0.049	0.42 ± 0.047 *	0.33 ± 0.029 *	0.34 ± 0.042	0.35 ± 0.034	0.35 ± 0.035	0.29 ± 0.028	0.27 ± 0.030	0.27 ± 0.033	0.25 ± 0.028	0.28 ± 0.025	0.35 ± 0.044	0.40 ± 0.064
Experiment – Collection latencies	**SN—DMS**	0.26 ± 0.015	0.28 ± 0.022	0.26 ± 0.017	0.27 ± 0.0086	0.28 ± 0.012	0.29 ± 0.029	0.26 ± 0.018	0.28 ± 0.033	0.26 ± 0.023	0.26 ± 0.008	0.25 ± 0.012	0.25 ± 0.011	0.25 ± 0.023	0.27 ± 0.028	0.25 ± 0.016	0.25 ± 0.013
	**VTA—NAcS**	0.28 ± 0.022	0.28 ± 0.029	0.28 ± 0.022	0.33 ± 0.072	0.42 ± 0.13	0.37 ± 0.12	0.27 ± 0.026	0.27 ± 0.015	0.27 ± 0.016	0.26 ± 0.028	0.25 ± 0.029	0.25 ± 0.020	0.26 ± 0.037	0.28 ± 0.037	0.31 ± 0.010	0.28 ± 0.0095

*Note*: Values given are means ± one standard error of the mean (s).

Abbreviations: DMS, dorsomedial striatum; SN, substantia nigra; VTA, ventral tegmental area.

* statistically significant differences reported.

#### 3.2.2 Lack of effect of SNc‐DMS pathway activation on reversal learning performance

**FIGURE 4:**
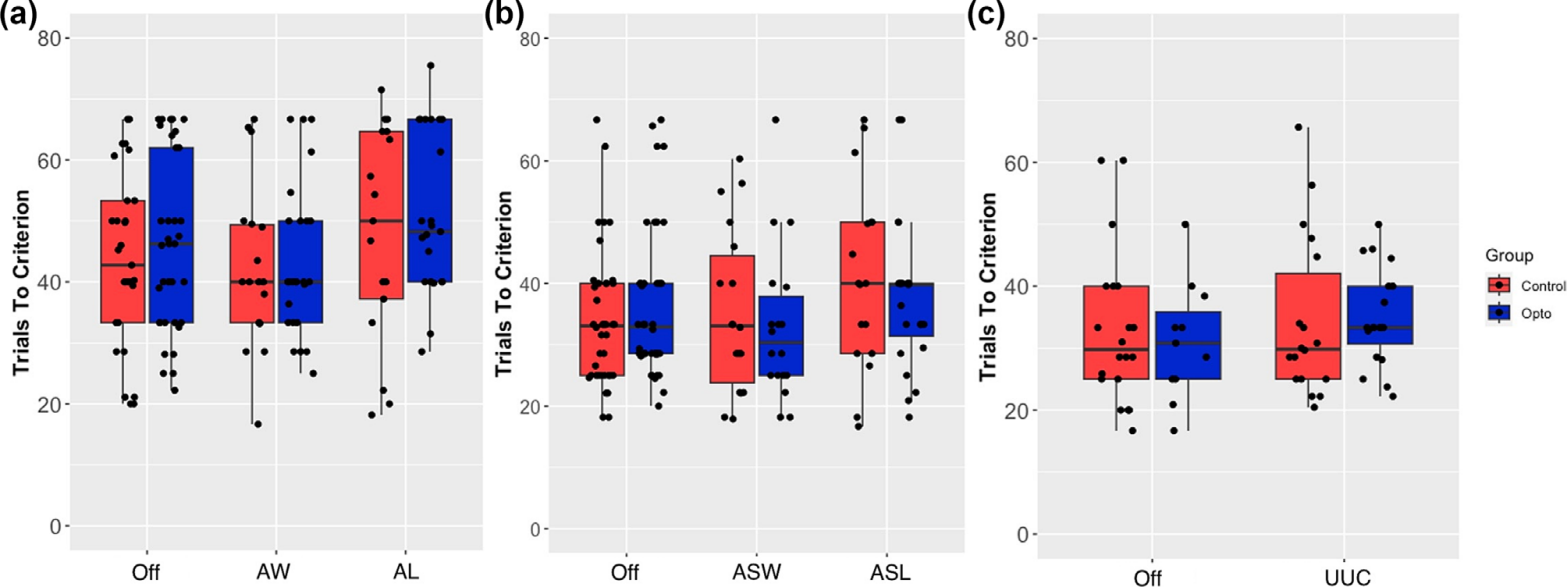
In vivo optogenetic stimulation of the nigrostriatal pathway has no significant effect on reversal learning performance. Trials to criterion required in each experiment: after win/after loss (*n* = 24) (a), after spurious win/after spurious loss (*n* = 23) (b), up until choice (*n* = 24) (c). The bars represent the range of data within 1.5 times the IQR from the lower and upper quartiles. AL, after loss; ASL, after spurious loss; AW, after win; ASW, after spurious win; Off, off light; UUC, up until choice.

In contrast to activation of the VTA‐NAcS pathway, activation of the SNc‐DMS pathway had no significant effect on TTC (Figure 4), number of reversals, lose‐shift, number of perseverative responses or any other measure in any of the experiments (AW/AL, ASW/ASL, UUC). There were also no significant group × condition interactions for the response or collection latencies for any of the experiments.

## 4 DISCUSSION

Using in vivo optogenetic stimulation, we report dissociable roles of the VTA‐NAcS and SNc‐DMS pathways in modulating the effects on reward feedback in a two‐choice spatial discrimination reversal task with probabilistic reinforcement. Whereas stimulation of the VTA‐NAcS pathway following reward omission (i.e., after loss of reward) impaired reversal learning performance by increasing the number of trials required to reach criterion, no such changes were observed after stimulation of the SNc‐DMS pathway. These findings are consistent with the hypothesis that hyperactivation of the VTA‐NAcS pathway impairs reversal learning specifically due to aberrant signals following reward omission, and that the NAcS and the DMS have dissociable roles in modulating reversal learning.

The dissociable effects of nigrostriatal DA stimulation are consistent with the NAcS and the DMS having dissociable but complementary roles in modulating reversal learning depending on the learning phase (Sala‐Bayo et al., 2020). The DMS is implicated in goal‐directed behaviours, making it necessary to develop and implement strategies to solve the task. In addition, a study employing a stimulus–response task identified the dorsal striatum as a key region involved in the initiation and execution of a learned instrumental response rather than the encoding of RPEs (Cox & Witten, 2019). Instead, the utilization of RPEs appear to be selectively processed by the NAcS, as suggested by the impairment related to negative feedback processing reported in the present study. Based on earlier studies (e.g., He et al., 2019; Peak et al., 2020), the SNc‐DMS pathway was predicted to interact with outcome evaluation and probabilistic reversal. However, photostimulation of this pathway had no significant effect on behaviour in our hands. These null findings do not necessarily rule out this pathway in PRL, but we acknowledge that our stimulation parameters may have been inadequate to engage a sufficient area of DMS to affect task performance. In addition, the substantial heterogeneity of DA release and uptake dynamics across different striatal subregions (e.g., Mohebi et al., 2024) challenges the view that RPEs are uniformly expressed across the striatum. Thus, the same stimulation parameters may not have been equally effective in the ventral and dorsal striatum in disrupting RPEs.

A further limitation of our study is that we cannot exclude a role for non‐dopaminergic neurons in the behavioural effects of VTA‐NAcS pathway stimulation. Although the opsin was mainly expressed in TH^+^ neurons, it was not restricted to these neurons and was also present in non‐TH^+^ neurons, including GAD67^+^ and VGLUT2^+^ neurons. The observed outcomes could thus result from the combined involvement of DA, GABA and glutamate. Indeed, although a convincing body of work has implicated DA in reinforcement learning, a recent study reported an additional contribution of glutamate from glutamate/DA co‐releasing VTA‐NAcS neurons in the reinforcing effects of optogenetic self‐stimulation (Zell et al., 2020). In addition, DA terminals co‐release glutamate preferentially in the ventromedial, but not the dorsal striatum (Mingote et al., 2019; Stuber et al., 2010; Tecuapetla et al., 2010). It is possible, therefore, that the observed effects following optogenetic stimulation of the NAcbS arose from the additional co‐modulation of reinforcement by released glutamate—perhaps explaining the lack of effect when targeting the DMS. More generally, our findings are in line with improved behavioural flexibility observed after inhibiting NAcS neurons (Aquili et al., 2014) and possibly also enhanced response‐switching induced by systemic d‐amphetamine administration (Evenden & Robbins, 1983). A further limitation of our study is that only male rats were included in this experiment. Sex‐dependent effects on reversal learning in rats have been reported previously (Chen et al., 2021; Evans & Hampson, 2015), but it is unknown how these differentially affect the VTA‐NAcS and SNc‐DMS pathways in the context of the present experimental intervention.

A small proportion of neurons co‐expressed the opsin virus and GAD67. Stimulation of VTA‐GABA projections to the NAc have been shown to enhance stimulus‐outcome learning, likely by inhibiting cholinergic neurons in the striatum (Brown et al., 2012). However, increased stimulus salience would result in improved performance, unlike the observed deficit in reversal learning. Hence, combined with the finding that GABA neurons were in a minority of neurons expressing the virus, it is unlikely the observed deficit in reversal learning was the result of increased GABA signalling in the NAcS.

A further consideration is the potential reinforcing effect of light applied via optogenetics, which could potentially exert rewarding effects. However, under similar conditions, the findings of Steinberg et al. (2013) refuted the possibility of conditioned reinforcing effects of the optogenetic stimulation. Additionally, when paired with reward (e.g., in rewarded trials, AW ‘after win’), the light could increase reward value as a conditioned reinforcer, leading to increased discriminability from the non‐rewarded stimulus. However, performance in reversal learning did not improve when either the mesolimbic or nigrostriatal pathways were stimulated during reward delivery; nor was there an increased value or preference for paired rewards (Steinberg et al., 2013). This suggests that optical stimulation was not sufficient to simulate the properties of the natural reward.

The present research expands and supports the role of the midbrain‐striatal circuit encoding RPEs as teaching signals to enable learning. For the first time, it demonstrates with temporal precision the link between hyperactivity within the VTA‐NAcS pathway and performance in reversal learning when negative outcomes are encoded. Our work adds to a burgeoning body of literature establishing and characterizing the causal link between DA signalling during RPEs and reward learning (Aquili, 2014; Chang et al., 2015; Steinberg et al., 2013). Further understanding of the processes involved in learning from negative feedback may be relevant for patients with major depressive disorder, schizophrenia or OCD, who show an accentuated bias towards negative feedback (Clark et al., 2009; Elliott et al., 1997; Hales et al., 2014).

## AUTHOR CONTRIBUTIONS

**Katharina Zühlsdorff:** Formal analysis; software; visualization; writing—original draft. **Júlia Sala‐Bayo:** Conceptualization; data curation; investigation; methodology; project administration; writing—original draft. **Sammy Piller:** Data curation; investigation; methodology. **Peter Zhukovsky:** Formal analysis; investigation; software. **Thorsten Lamla:** Conceptualization; investigation; methodology; resources. **Wiebke Nissen:** Investigation; methodology; project administration. **Moritz von Heimendahl:** Conceptualization; data curation; investigation; project administration; resources; supervision. **Serena Deiana:** Conceptualization; investigation; methodology. **Janet R. Nicholson:** Conceptualization; investigation; resources; supervision. **Trevor W. Robbins:** Conceptualization; funding acquisition; supervision. **Johan Alsiö:** Conceptualization; formal analysis; investigation; methodology; project administration; software; supervision; validation; writing—original draft. **Jeffrey W. Dalley:** Conceptualization; funding acquisition; methodology; resources; supervision; writing—original draft.

## CONFLICT OF INTEREST STATEMENT

The remaining authors declare no conflict of interest.

### PEER REVIEW

The peer review history for this article is available at https://www.webofscience.com/api/gateway/wos/peer-review/10.1111/ejn.16584.

## Data Availability

The data that support the findings of this study are available from the corresponding author upon request.
